# Abnormal body weight and risk of gallbladder cancer: an updated meta-analysis incorporating recent evidence

**DOI:** 10.1186/s12885-026-16320-8

**Published:** 2026-06-23

**Authors:** Wooyoung Chung, Minseok Kang, Jeong Min Sung, Kyu Nam Kim, Ji Yoon Choi, Yun Kyung Jung, Dongho Choi

**Affiliations:** 1 Department of Surgery, Hanyang University College of Medicine, Seoul, 04763 Republic of Korea; 2 Hanyang Institute of Bioscience and Biotechnology, Hanyang University, Seoul, Republic of Korea; 3 Department of HY-KIST Bio-Convergence, Hanyang University, Seoul, Republic of Korea; 4 Department of Anesthesiology and Pain Medicine, College of Medicine, Hanyang University Hospital, Seoul, Republic of Korea

**Keywords:** Gallbladder Cancer, Body Mass Index, Excess body weight, Abnormal body weight, Overweight, Obesity, Underweight

## Abstract

**Background:**

To update previous meta-analyses by evaluating the association between body mass index (BMI) categories (underweight, overweight, and obesity) and gallbladder cancer (GBC) risk, and to compare findings between earlier and more recent studies.

**Methods:**

A systematic search of PubMed and Web of Science identified 10 observational studies published between January 2017 and May 2025. Studies reporting risk ratios (RRs), hazard ratios, or odds ratios with 95% confidence intervals (CIs) for GBC by BMI category were included. Pooled RRs were calculated using fixed- and random-effects models, and heterogeneity was assessed using I^2^ statistics. Subgroup analyses compared older with newer studies, and men with women.

**Results:**

Twenty-four studies (14 previous, 11 new) were included. The underweight group showed a a 5% increased possible risk of GBC (fixed-effects RR = 1.05, 95% CI: 0.89–1.24), but there was no clear evidence as statistical significance was not demonstrated. The overweight group was associated with a 19% increased risk (random-effects RR = 1.19, 95% CI: 1.11–1.27), and obesity with a 69% increased risk (random-effects RR = 1.69, 95% CI: 1.51–1.88). Recent studies reported higher RRs for the overweight and obesity groups compared with earlier studies.

**Conclusions:**

Excess body weight is significantly associated with increased GBC risk, with stronger associations reported in recent studies. Underweight also suggests a possible positive association. These findings emphasize the importance of weight management in GBC prevention strategies.

**Trial registration:**

Prospero registration ID: CRD420251089832.

**Supplementary Information:**

The online version contains supplementary material available at https://doi.org/10.1186/s12885-026-16320-8.

## Introduction

Gallbladder cancer (GBC) is the sixth most common cancer worldwide and the most common malignancy to develop in the biliary system [1]. According to the GLOBOCAN Annual Report in 2010, 115,000 individuals were diagnosed with GBC globally, accounting for 0.6% of all cancers, and 84,000 died from the disease, representing 0.63% of all cancer-related deaths [2, 3]. The high mortality associated with GBC is attributable to its unique characteristics. Early detection of GBC can increase the five-year survival rate to over 80% [4]. Unfortunately, due to its malignant nature, delayed presentation, complex anatomic location, and advanced stage at diagnosis, the prognosis for GBC remains poor [1]. These challenges result in a 5-year survival rate of approximately 5% and a median survival time of only 6 months [5]. Reported risk factors for GBC include gallstones [6], porcelain gallbladder [7], gallbladder polyps [8], chronic infection [9–11], obesity [12], and diabetes [13].

Obesity, a well-recognized risk factor for GBC, has emerged as a major global health challenge, profoundly affecting both public health and individual outcomes. Its prevalence has risen markedly, placing increasing strain on healthcare systems worldwide [14]. According to a World Health Organization (WHO) report in 2023, the global prevalence of obesity has nearly doubled since 1980 [14]. Similarly, it was predicted in the 2023 report of the World Obesity Federation that more than half of the global population will be living with obesity or being overweight within the next 12 years [14]. Of particular concern is the strong association between obesity and cancer risk. Growing evidence demonstrates a robust and consistent link between obesity and increased susceptibility to multiple cancers, including GBC [15].

Several meta-analyses have examined the relationship between excess body weight and the risk of GBC. However, the number of studies directly addressing this association remains limited. Although Piovani et al. [16] reported a correlation between excess body weight and GBC risk, the topic was not explored in depth. Furthermore, no new meta-analyses focusing specifically on body weight and GBC risk have been published since the 2016 reports by Li et al. [17] and Liu et al. [18]. The studies included in those meta-analyses are relatively old, limiting their ability to reflect the current evidence landscape. In addition, although some studies in Liu et al. [18] indicated an elevated GBC risk among underweight individuals, this finding was overlooked, with analyses restricted to overweight and obese groups. Therefore, updating the available evidence with recently conducted studies is necessary to better define the relationship between body weight and GBC risk. Accordingly, it is sought to re-establish association between non-normal body weight and the risk of GBC by defining underweight (BMI < 18.5 kg/m^2^), overweight (BMI 25–29.9 kg/m^2^), and obesity (BMI ≥ 30 kg/m^2^), based on the WHO classification, as “abnormal body weight”, which is out of normal body weight (BMI 18.5–24.9 kg/m^2^).

## Methods

### Study design

This meta-analysis was conducted in accordance with the Meta-analysis Of Observational Studies in Epidemiology (MOOSE) guidelines [19]. Our study followed the PRISMA 2020 statement [20]. The PRISMA checklist was also completed (Table S1). The protocol for the study has been registered in PROSPERO with the following ID number: CRD420251089832.

### Search strategy

To update prior analyses of Li et al. [17] and Liu et al. [18], including 14 common studies, a comprehensive search was performed for studies published between January 2017 and May 2025. Studies were collected from two databases: PubMed and Web of Science. The following keywords were used: “body mass index” OR “BMI” OR “obesity” OR “overweight” OR “underweight” combined with “gallbladder cancer” OR “gallbladder carcinoma.” The search was restricted to the studies conducted in humans and published in English.

### Inclusion and exclusion criteria

Two investigators independently evaluated all retrieved studies to assess eligibility. Disagreements were resolved through discussion and, when necessary, consultation with the senior author. Studies were included if they met the following criteria: (1) full-text articles accessible in the database; (2) exposure of interest was underweight, overweight, or obesity defined by BMI or by diagnosis at discharge; (3) the study reported an association between BMI and GBC; and (4) the association was expressed as a risk ratio (RR), odds ratio (OR), or hazard ratio (HR) with the corresponding confidence interval (CI).

Studies were excluded if: (1) they were not full reports (e.g., conference abstracts or letters to the editor); (2) the full text was not freely available in the database; (3) RRs, ORs, or HRs were reported only for specific BMI ranges; or (4) they lacked sufficient calculations for data synthesis.

### Data extraction

The following information was extracted from each study: the last name of the first author (with “et al.” added if more than three authors), year of publication, study design, country, follow-up duration (for cohort studies), age range or mean baseline age, sex, number of participants, outcome, HR/OR/RR with 95% CIs, and variables adjusted in the analysis. When studies reported multiple types of adjusted risks (e.g., univariate and multivariate), the most fully adjusted estimate was extracted.

### Quality assessment

The quality of selected studies was evaluated using the Risk of bias in non-randomized studies of interventions (ROBINS-I) tool. ROBINS-I assesses the validity and potential risk of bias in effect estimates from non-randomized studies [21]. It evaluates seven domains: confounding, selection of participants, classification of interventions, deviations from intended interventions, missing data, measurement of outcomes, and selection of reported results. Each domain was graded as “Low,” “Moderate,” “Serious,” or “Critical.” Although quality assessment of the 14 previously included studies had already been performed using the Newcastle–Ottawa Scale (NOS) in earlier meta-analyses, since NOS and ROBINS-I are not directly comparable tools, the 14 studies utilized in the previous meta-analyses were also subjected to a quality assessment using ROBINS-I; therefore, reassessment was conducted for those studies [17, 18].

### Statistical analysis

Random-effects and fixed-effects meta-analyses were used to estimate the summary RR for the association between BMI and GBC risk. In this analysis, as GBC is a rare disease with a prevalence of less than 10%, HRs and ORs were considered equivalent to RRs, in accordance with the rare disease rule [22]. Following the WHO classification for BMI in adults, summary RRs were calculated for underweight (BMI < 18.5 kg/m^2^), overweight (BMI 25–29.9 kg/m^2^), and obesity (BMI ≥ 30 kg/m^2^) using normal weight (BMI 18.5–24.9 kg/m^2^) as the reference category. When nonstandard BMI categories were reported, the categories most similar to the WHO definitions were selected [23]. Additionally, the RR per 5 kg/m^2^ increment in BMI was estimated using the generalized least-squares method for trend estimation [24]. The median or mean in each BMI category was assigned to its corresponding dose. If not reported, the midpoint of the upper and lower boundaries of the category was used. For open-ended highest categories, the median value was assumed as the lower cut-off plus a 25% increment [18]. Subgroup analyses were performed to compare results from studies included in previous meta-analyses with those from newly selected studies, as well as to compare results based on sex. These analyses aimed to determine whether the association between BMI and GBC risk differed between earlier and more recent evidence. Funnel plot and Egger’s regression test were also used to check the publication bias. Furthermore, the analysis of results based on sex aims to determine which sex constitutes a higher-risk group and to identify the associated factors. Statistical heterogeneity was assessed using the I^2^ statistic, with thresholds of 25%, 50%, and 75% indicating low, moderate, and high heterogeneity, respectively [25]. Fixed-effects models were applied when heterogeneity was < 50%, while random-effects models were applied when heterogeneity exceeded 50%. Sensitivity analyses were conducted to evaluate the robustness of the pooled effect estimates. Analyses were repeated after excluding studies assessed as having a serious risk of bias according to the ROBINS-I v2 tool. This approach was used to determine whether the inclusion of lower-quality studies influenced the overall results. In addition, a leave-one-out sensitivity analysis was performed, in which each study was sequentially removed and the pooled estimate recalculated to assess the influence of individual studies on the overall findings. Particular attention was given to the exclusion of the study with the highest weight to evaluate whether the results were disproportionately driven by highly weighted studies. All statistical analyses were performed using R Studio.

## Results

### Literature search

**Fig. 1:**
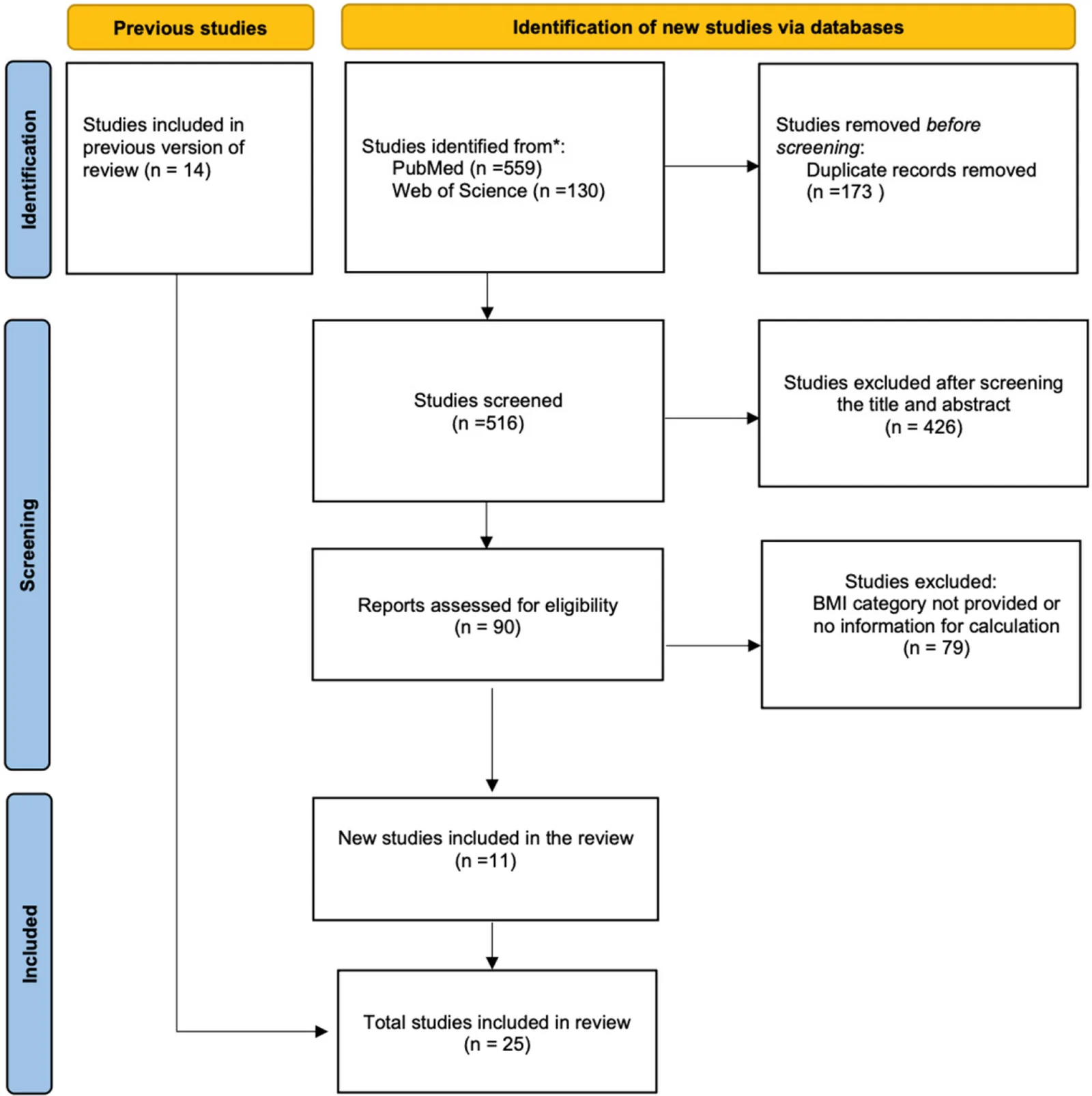
Flowchart of study selection. Flow chart illustrating the literature search for studies on BMI in relation to GBC

The study selection process is shown in Fig. 1. A total of 689 studies were initially extracted, 559 from PubMed and 130 from Web of Science. Of these, 173 were excluded as duplicates. The remaining 516 studies were screened by titles and abstracts, resulting in the exclusion of 426. Eligibility was assessed for 90 studies, and after full-text review, 79 were excluded because they did not report RRs with 95% CIs or did not provide sufficient data for calculation. Ultimately, 14 previous studies [26–39] and 11 newly identified studies [40–50] were included in the meta-analysis (Table 1).

**Table 1:** Characteristics of included studies in the meta-analysis

Study ID, Country	Study design	No. Case	Age: mean or range	Follow-up (Year) mean or range	BMI categories (kg/m2)	RR and 95% CI			Adjustment	Quality assessment: ROBINS-1 v2
						Total	Men	Women		
Kato et al., 1992 [38] USA	Cohort	Total: 90 Men: N/A Women: N/A	Born from 1900 to 1919	≥ 22	< 21.65 21.65–23.19 23.80–25.80 > 25.80	N/A	1.0 1.1 (0.9–1.5) 1.4 (1.1–1.9) 1.8 (1.4–2.3)	N/A	Occupation, education, smoking, dietary, alcohol, age, physical activity	Moderate
Møller et al., 1994 [27] Denmark	Cohort	Total: 28 Men: N/A Women/A	Women:50 Men:60	5	Non-obese Obese	1.0 (reference) 1.30 (0.8–1.8)	1.0 (reference) 0.50 (0.1–1.8)	1.0(reference) 1.40 (0.9–2.1)	Age	Moderate
Wolk et al., 2001 [28] Sweden	Cohort	Total: 31 Men: N/A Women: N/A	46.1	10.3	Non-obese Obese	1.0(reference) 1.60 (1.1–2.3)	1.0(reference) 0.90 (0.1–3.4)	1.0(reference) 1.70 (1.1–2.5)	Age, calendar year	Moderate
Calle et al., 2003 [39] USA	Cohort	Total: 484 Men: N/A Women: N/A	57	16	18.5–24.9 25.0–29.9 30.0–34.9 ≥ 35	N/A	1.00 (reference) 1.34 (0.97–1.84) 1.76 (1.06–2.94) N/A	1.0(reference) 1.12 (0.86–1.47) 2.13 (1.56–2.90) N/A	Age, race, marital status, smoking, aspirin, alcohol, estrogen therapy	Low
Samanic et al., 2004 [40] USA	Cohort	Total: 338 Men: 291 Women:47	White:52.18 Women:47.63	1–27	Non-obese Obese	1.0 (reference) 1.62 (1.09–2.41)	1.0(reference) White: 0.93 (0.23–3.86) Black: 1.70 (1.13–2.57)	N/A	Age, calendar year	Moderate
Kuriyama et al., 2005 [32] Japan	Cohort	Total: 33 Men: N/A Women: N/A	≥ 40	9	< 18.5 18.5–24.9 25.0–29.9 ≥ 30.0	N/A	1.00 (reference) 0.46 (0.05–3.93) N/A N/A	1.00 (reference) 0.83 (0.23–2.98) 3.43 (1.19–9.94) 4.45 (1.39–14.23)	Age, smoking, health insurance, alcohol	Low
Oh et al., 2005 [32] Korea	Cohort	Total:182 Men:N/A Women:N/A	≥ 20	10	< 18.5 18.5–22.9 23.0–24.9 25.0–26.9 27.0–29.9 ≥ 30.0	N/A	2.44 (1.12–5.34) 1.00 (reference) 1.5 (1.10–2.20) 1.1 (0.74–1.80) 1.2 (0.70–2.24) N/A	N/A	Age, smoking, alcohol, exercise, region	Low
Anders England et al., 2005 [29] Norway	Cohort	Total: 1715 Men: N/A Women: N/A	44 (20–74)	23	< 18.5 18.5–24.9 25.0–29.9 ≥ 30.0	N/A	0.31 (0.04–2.24) 1.00 (reference) 1.00 (0.84–1.17) 1.38 (1.01–1.89)	1.02 (0.54–1.91) 1.00 (reference) 1.27 (1.10–1.47) 1.88 (1.60–2.21)	Age, birth	Moderate
Samanic et al., 2006 [30] Sweden	Cohort	109 Men: N/A Women: N/A	18–67	28	25.0–29.9 ≥ 30.0	N/A	0.93 (0.62–1.39) 1.40 (0.73–2.70)	N/A	Age, smoking	Low
Ishiguro et al., 2007 [34] Japan	Cohort	93 Men: N/A Women: N/A	40–69	10.9	≤ 22.9 23.0–24.9 25.0–26.9 ≥ 27.0	N/A	1.00 (reference) 0.74 (0.28–1.92) 1.26 (0.48–3.33) 1.39 (0.45–4.34)	1.00 (reference) 0.47 (0.22–0.98) 0.62 (0.29–1.34) 0.94 (0.48–1.88)	Age, gender, study area, diabetes, smoking, alcohol	Moderate
Jee et al., 2008 [35] Korea	Cohort	1882 Men: N/A Women: N/A	Men: 45.0 Women: 49.4	10.8	25.0–29.9 ≥ 30	1.00 (0.89–1.12) 1.54 (1.17–2.03)	0.97 (0.86–1.10) 1.65 (1.11–2.44)	1.27 (1.02–2.12) 1.44 (0.98–2.12)	Age, smoking, alcohol, physical activity	Low
Song et al., 2008 [37] Korea	Cohort	181 Men: N/A Women: N/A	40–64 (55.9)	8.75	< 18.5 18.5–20.9 21.0–22.9 23.0–24.9 25.0–26.9 27.0–29.9 ≥ 30	N/A	N/A	1.91 (0.78–4.68) 1.35 (0.74–2.47) 1.00 (reference) 1.06 (0.62–1.80) 1.30 (0.76–2.22) 1.86 (1.09–3.18) 2.10 (0.97–4.51)	Age, height, smoking, alcohol, exercise, pay level	Low
Hemminki et al., 2011 [31] Sweden	Cohort	19 Men: N/A Women: N/A	N/A	11.2	Non-obese obese	1.73 (1.16–2.57)	N/A	1.55 (0.93–2.43)	Age, sex, region, economic status	Moderate
Schlesinger et al., 2013 [26] Europe	Cohort	75 Men: N/A Women: N/A	25–70	8.5	Non-obese obese	2.71 (1.17–6.31)	N/A	N/A	Weight, height, waist circumference, alcohol, smoking, education, diet, lifestyle, medical history, blood samples	Moderate
Campbell et al., 2017 [42] international	Cohort	Total:567 Men:160 Women:407	56.7	N/A	< 18.5 18.5–24.9 25–29.9 30–34.9 35–39.9 ≥ 40.0	1.21(0.60–2.47) 1.00(reference) 1.27(1.04–1.54) 1.53(1.18–1.98) 1.86(1.25–2.78) 2.31(1.30–4.09)	2.03(0.49–8.50) 1.00(reference) 1.46(0.99–2.16) 2.11(1.30–3.44) 1.39 (0.49–3.96) 2.61 (0.62–11.0)	1.06(0.47–2.39) 1.00(reference) 1.21(0.97–1.52) 1.35(0.99–1.83) 1.97(1.28–3.05) 2.28(1.22–4.26)	Age Sex Race Education, Smoking status Alcohol consumption, Physical activity (History of gallstones)	Moderate
Zakaria & Shaw 2017 [50] Canada	Cohort	Total:110 Men:35 Women:75	≥ 25	N/A	18.5–24.9 25.0–29.9 ≥ 30.0	N/A	1.00(reference) 1.24(1.11–1.39) 1.52(1.23–1.88)	1.00(reference) 1.31(1.06–1.51) 1.67(1.11–2.23)	sex, region	Low
Jeong et al., 2018 [36] Korea	Cohort	Total:324 Men:N/A Women:N/A	N/A	10.5	< 18.5 18.5–24.9 25–29.9 ≥ 30.0 < 18.5(NS) 18.5–24.9(NS) 25.0–29.9(NS) ≥ 30.0(NS)	1.47(0.86–2.50) 1.00(reference) 1.17(0.92–1.48) 1.91(1.14–3.20) 1.52 (0.74–3.13) 1.00(reference) 1.30 (0.97–1.74) 2.44 (1.42–4.19)	N/A	N/A	Age Sex Smoking	Moderate
Lee et al., 2019 [46] Korea	Cohort	Total:98 Men:27 Women:71	40–79	N/A	< 18.5 18.5–22.9 23.0–24.9 25.0–29.9 ≥ 30.0	N/A	0.85(0.49–1.47) 1.00(reference) 1.09 (0.89–1.32) 0.94 (0.77–1.14) 1.25 (0.75–2.09)	1.51(0.93–2.46) 1.00(reference) 1.05(0.84–1.31) 1.26(1.03–1.54) 1.50 (1.02–2.19)	age, income, total cholesterol, family history of cancer, Charlson comorbidity index, Smoking status, Alcohol consumption, Physical activity, Regular use of aspirin	Low
Jackson et al., 2019 [45] international	Cohort	Total:1343 Men:N/A Women:N/A	57	18	< 18.5 18.5–24.9 25.0–29.9 ≥ 30.0	0.98(0.60–1.60) 1.00(reference) 1.31(1.10–1.54) 1.72 (1.41–2.08)	N/A	N/A	Age, Sex, Race, Education, level, Smoking, Alcohol consumption, History of gallbladder disease	Moderate
Fujii et al.,2019 [44] Japan	Cohort	Total:450 Men:N/A Women:N/A	74	N/A	< 18.5 18.5–24.9 ≥ 25.0	1.00(reference) 1.14 (0.85–1.53) 1.09 (0.77–1.53)	N/A	N/A	Age Sex Cancer Stage Operation Method	Moderate
Recalde et al., 2021 [47] Spain	Cohort	Total:1355 Men:N/A Women:N/A	36	7.7	18.5–24.9 25.0–29.9 ≥ 30.0 18.5–24.9(NS) 25.0–29.9(NS) ≥ 30.0(NS)	1.00(reference) 1.12(1.05–1.19) 1.25(1.10–1.41) 1.00(reference) 1.19(1.08–1.25) 1.41(1.17–1.56)	N/A	N/A	Age Sex Smoking status	Moderate
Sun et al., 2023 [49] UK, USA, China	Cohort	Total:504 Men:N/A Women:N/A	42.8	19.7	18.5–24.9 25.0–29.9 ≥ 30.0 18.5–24.9(UH) 25.0–29.9(UH) ≥ 30.0(UH)	1.00(reference) 1.45 (1.12–1.89) 1.84 (1.15–2.94) 1.27 (0.94–1.71) 1.37 (1.05–1.79) 1.62 (1.17–2.26)	N/A	N/A	Age Sex Smoking status Physical activity Alcohol consumption	Serious
Saeed et al., 2023 [48]	Cohort	Total:832 Men:176 Women:656	49.1	22.9	18.5–24.9 25.0–29.9 ≥ 30.0 18.5–24.9(YA) 25.0–29.9(YA) ≥ 30.0(YA)	1.00(reference) 1.53(1.40–1.68) 2.19(1.85–2.60) 1.00(reference) 1.65(1.20–2.26) 2.82(1.53–5.20)	1.00(reference) 1.11 (0.81–1.53) 1.26 (0.70–2.29) 1.00(reference) 1.34 (0.54–3.32) 1.86 (0.35–9.93)	1.00(reference) 1.56 (1.42–1.72) 2.42 (2.01–2.91) 1.00(reference) 1.70 (1.20–2.42) 2.93 (1.52–5.65)	Age, sex, time period	Moderate
Alkhayyat et al., 2021 [41] USA	Cohort	Total:4790 Men:1770 Women:3020	≥ 30	N/A	Non-Obese Obese	1.00(reference) 3.30(3.08–3.54)	N/A	N/A	Age Sex Race/ethnicity Region Year of diagnosis Socioeconomic indicators	Moderate
Dikaiou et al., 2024 [43] Sweden	Cohort	Total:281 Men:0 Women:281	49.7	16.3	< 18.5 18.5–19.9 20.0–22.4 22.5–24.9 25.0–29.9 ≥ 30.0	N/A	N/A	0.89(0.43–1.82**)** 0.80(0.51–1.25) 1.0(reference) 1.12(0.83–1.51) 1.02(0.71–1.45) 2.06(1.34–3.16)	Age Race/ethnicity	Moderate

*NS* Non-smoker group, *UH* Unhealthy group, *YA* Young age group, *ROBINS-I v2* Risk of bias in non-randomized studies of interventions

The table should be located directly below Fig. 1 during production

### Study characteristics

In total, 25 studies published between 1992 and 2024 were included. All studies examined the association between abnormal body weight and GBC. Of these, 16 studies reported associations between GBC and obesity within the WHO-defined BMI range, 14 reported associations between GBC and overweight, and 10 reported associations between GBC and underweight. 14 studies reported results for both sexes combined, nine reported results stratified by sex, four reported results for men only, and three reported results for women only. Quality assessment showed that, among the 25 included studies evaluated with the ROBINS-I v2 tool, eight were graded as low risk of bias, 16 as moderate, and one as serious.

### Publication bias

**Fig. 2:**
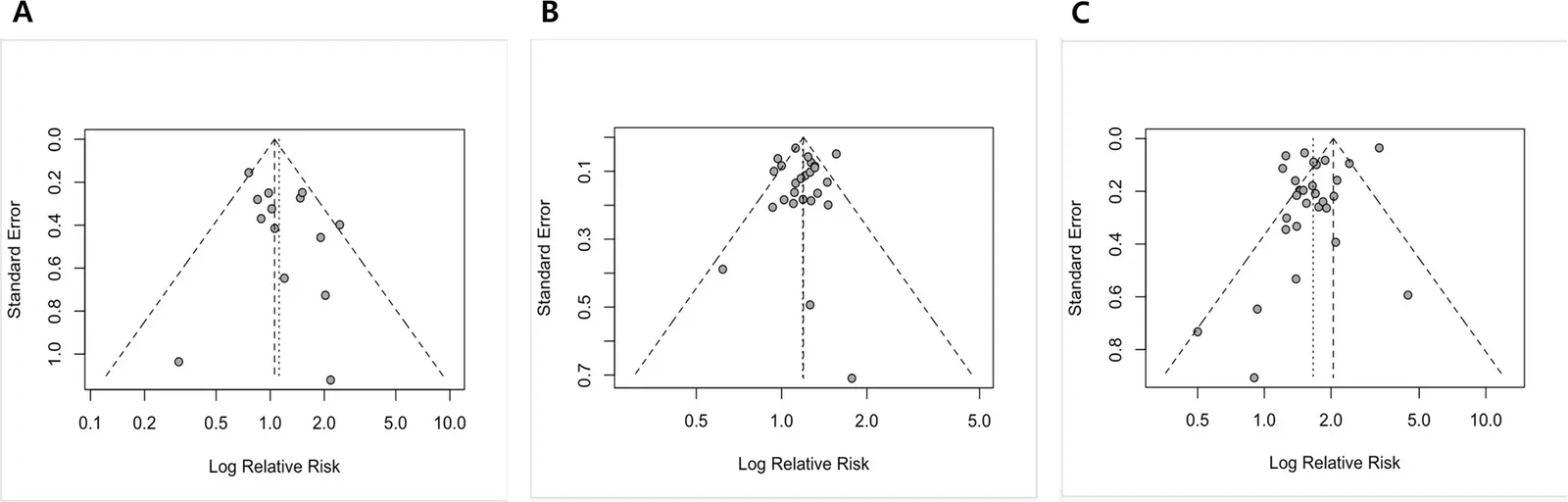
Publication Bias of Underweight, Overweight, and Obese Groups using Funnel Plot. **A** Underweight group, **B** Overweight group, **C** Obese group

**Fig. 3:**
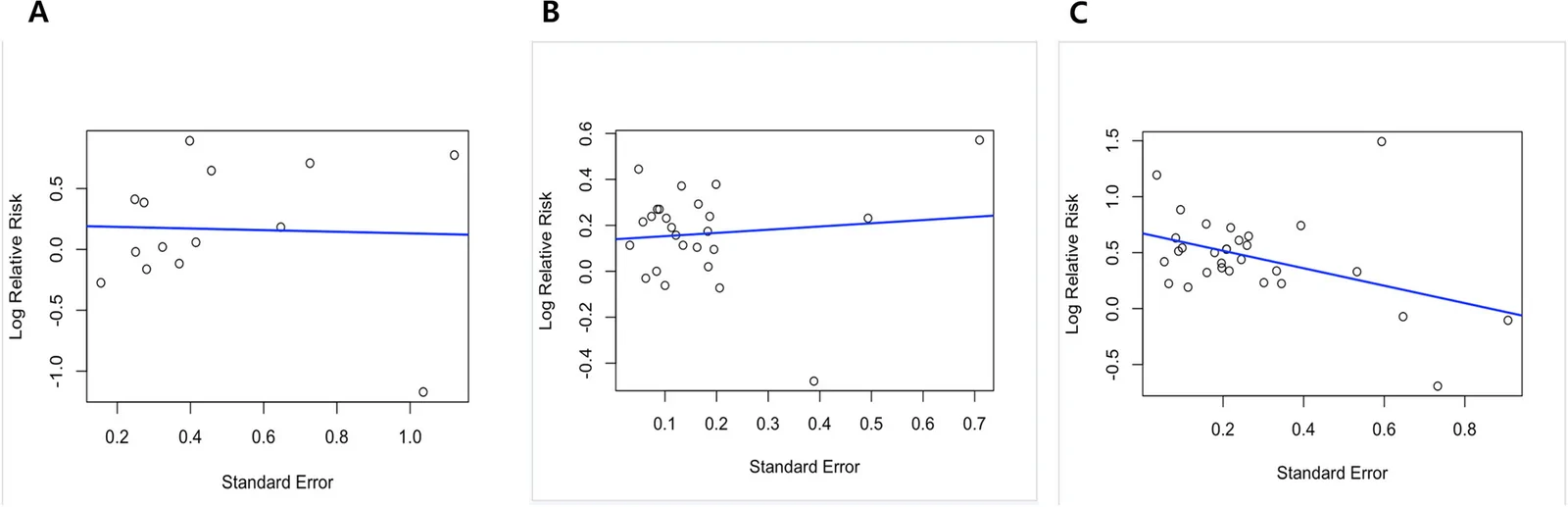
Publication Bias of Underweight, Overweight, and Obese Groups using Egger Regression Test. **A** Underweight group, **B** Overweight group, **C** Obese group

Two methods, the funnel plot and Egger’s regression test, were used to investigate the possibility of publication bias. Publication bias was analyzed by separately examining studies on underweight, overweight, and obese groups. For the underweight group, the funnel plot appeared generally symmetrical, although slight asymmetry was observed (Fig. 2A). In the overweight group, the funnel plot showed a largely symmetrical distribution, suggesting no substantial evidence of publication bias (Fig. 2B). In the obese group, asymmetry was observed as studies exhibited a tendency to skew toward the left (Fig. 2C). It indicated the possibility that only statistically significant results had been published. Consequently, the possibility that the actual effect was overestimated was taken into consideration. Meanwhile, Egger’s regression test revealed no evidence of publication bias in any of the groups (Underweight group: z = 1.0390, *p* = 0.2988, Overweight group: z = −0.5442, *p* = 0.5863, Obese group: z = −1.4738, *p* = 0.1405) (Fig. 3).

### Sensitivity analysis

**Fig. 4:**
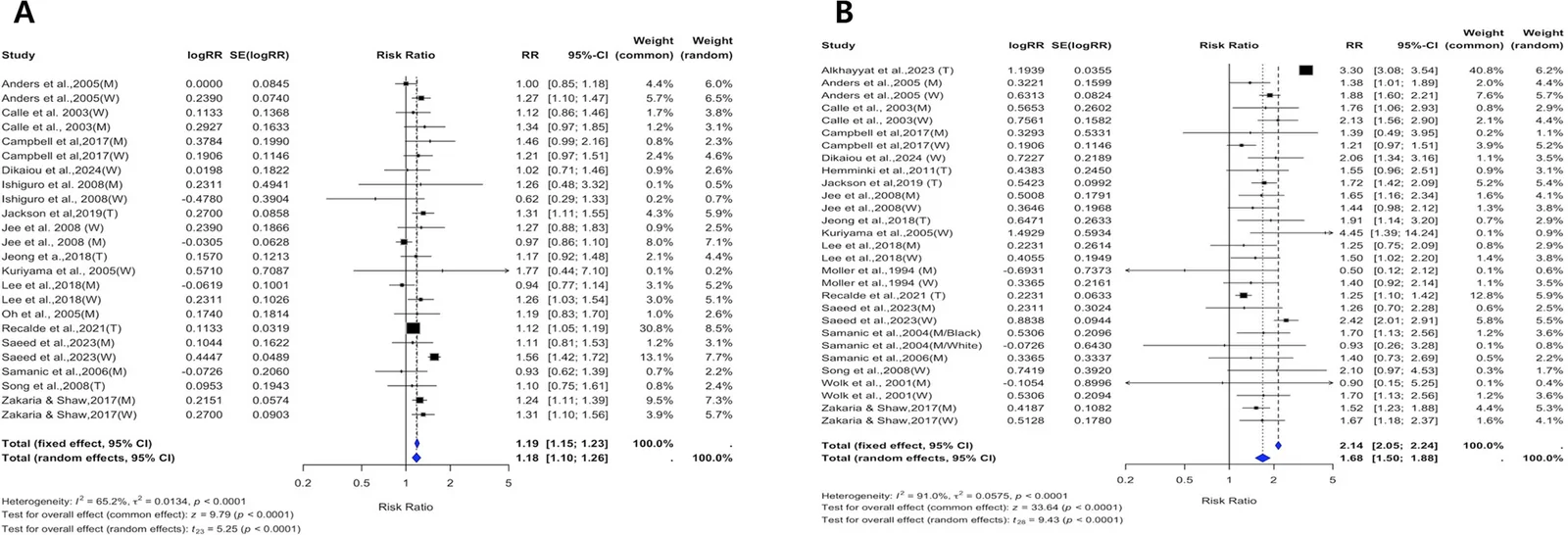
Sensitivity Analysis excluding studies with serious risk. **A** Overweight group, **B** Obese group, M: Men, W: Women, T: Total

The sensitivity analysis was conducted while excluding the study by Sun et al. [49], which was determined to carry a serious risk of bias. Since the study by Sun et al. included only the overweight and obese groups, the sensitivity analysis was performed within these two groups. The results remained materially unchanged compared with the original analysis, with no meaningful differences in the pooled effect estimates or their statistical significance (Fig. 4). This suggests that the overall findings were not unduly influenced by studies with a higher risk of bias.

### Leave-one-out analysis

**Fig. 5:**
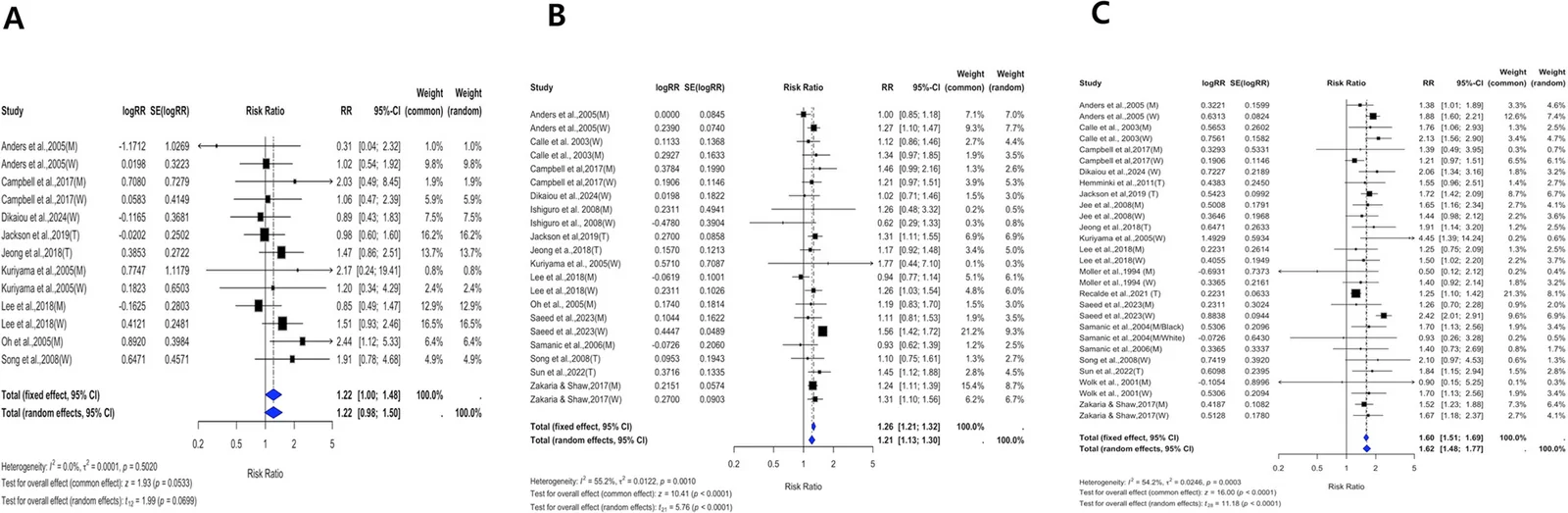
Leave-one-out analysis excluding a study with the largest sample size. **A** Underweight Group, **B** Overweight group, **C** Obese group. M: Men, W: Women, T: Total

The leave-one-out analysis excluded the study results with the highest weight within each of the underweight, overweight, and obese groups. In the underweight group, the study by Fujii et al. [44] was excluded; in the overweight group, the study by Recalde et al. [47] was excluded; and in the obese group, the study by Alkhayyat et al. [41] was excluded. The analysis of the underweight group indicated that a study by Fujii et al. [44] influenced the overall results (Fig. 5A). In contrast, the pooled effect estimates remained largely unchanged, with no meaningful differences in magnitude or statistical significance in the overweight and obese analysis (Fig. 5B, C). These findings indicate that the overall results of the overweight and obese groups were not driven by any single study, including the most influential study by sample size.

### Association between underweight and GBC risk

**Fig. 6:**
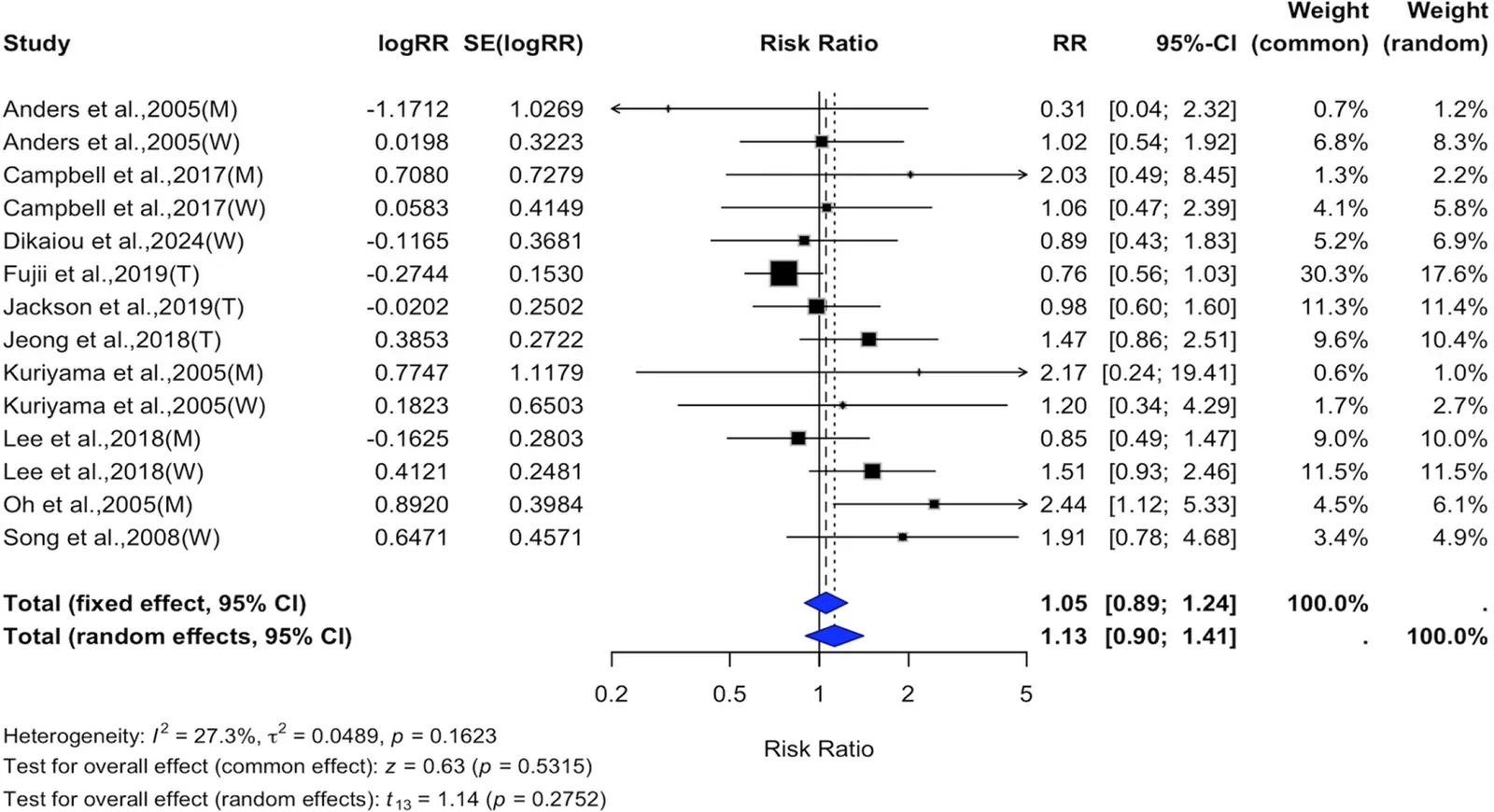
Relative risks of gallbladder cancer associated with BMI. Forest plot of the risk of GBC associated with underweight (< 18.5 kg/m2) in the general population. M: Men, W: Women, T: Total

Fourteen reports from 10 studies examined the association between GBC risk and underweight. As shown in Fig. 6, the pooled RR under the fixed-effects model was modestly elevated at 1.05 (95% CI: 0.89–1.24), while the random-effects model produced a slightly higher estimate of 1.13 (95% CI: 0.90–1.41). These findings suggest that although individuals classified as underweight may have a 5–13% increased risk of developing GBC compared to those with normal BMI, this increase was not statistically significant and therefore does not provide clear evidence of an association. The observed heterogeneity was low to moderate across studies (I^2^ = 27.3%, *p* = 0.1623), indicating a notable degree of inter-study variability; therefore, the fixed-effects model was selected for the primary analysis. Tests for the overall effect were not significant (z = 0.63, *p* = 0.5315 for the fixed-effects model; t = 1.14, *p* = 0.2752 for the random-effects model).

### Association between overweight and GBC risk

**Fig. 7:**
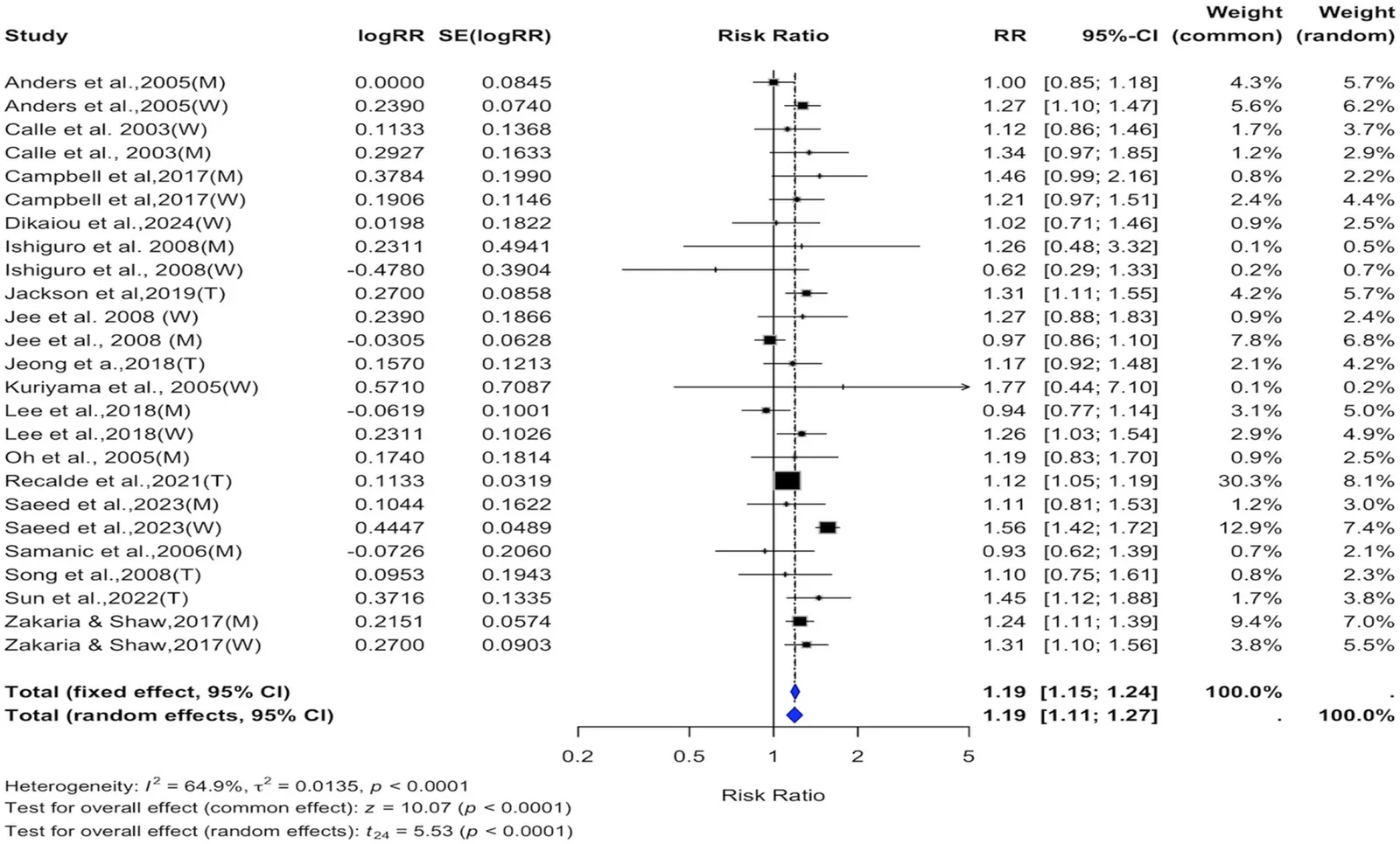
Relative risks of gallbladder cancer associated with BMI. Forest plot of the risk of GBC associated with overweight (25–29.9 kg/m2) in the general population. M: Men, W: Women, T: Total

Twenty-five reports from 16 studies assessed the association between GBC risk and overweight. Figure 7 represents a forest plot illustrating the results of a meta-analysis examining the relationship between being overweight and the risk of GBC. The pooled RR derived from both fixed-effects and random-effects models was 1.19, with corresponding 95% CI of 1.15–1.24 and 1.11–1.27, respectively. These results indicate a statistically significant 19% increased risk of GBC among overweight individuals compared with those of normal BMI. Heterogeneity was moderate to high (I^2^ = 64.9%, *p* < 0.0001), suggesting notable inter-study variability; therefore, a random-effects model was applied in the results analysis. The consistency in effect size across models supports the robustness of the association. Tests for overall effect were highly significant (z = 10.07, *p* < 0.0001 for the fixed-effects model; t = 5.53, *p* < 0.0001 for the random-effects model).

### Association between obesity and GBC risk

**Fig. 8:**
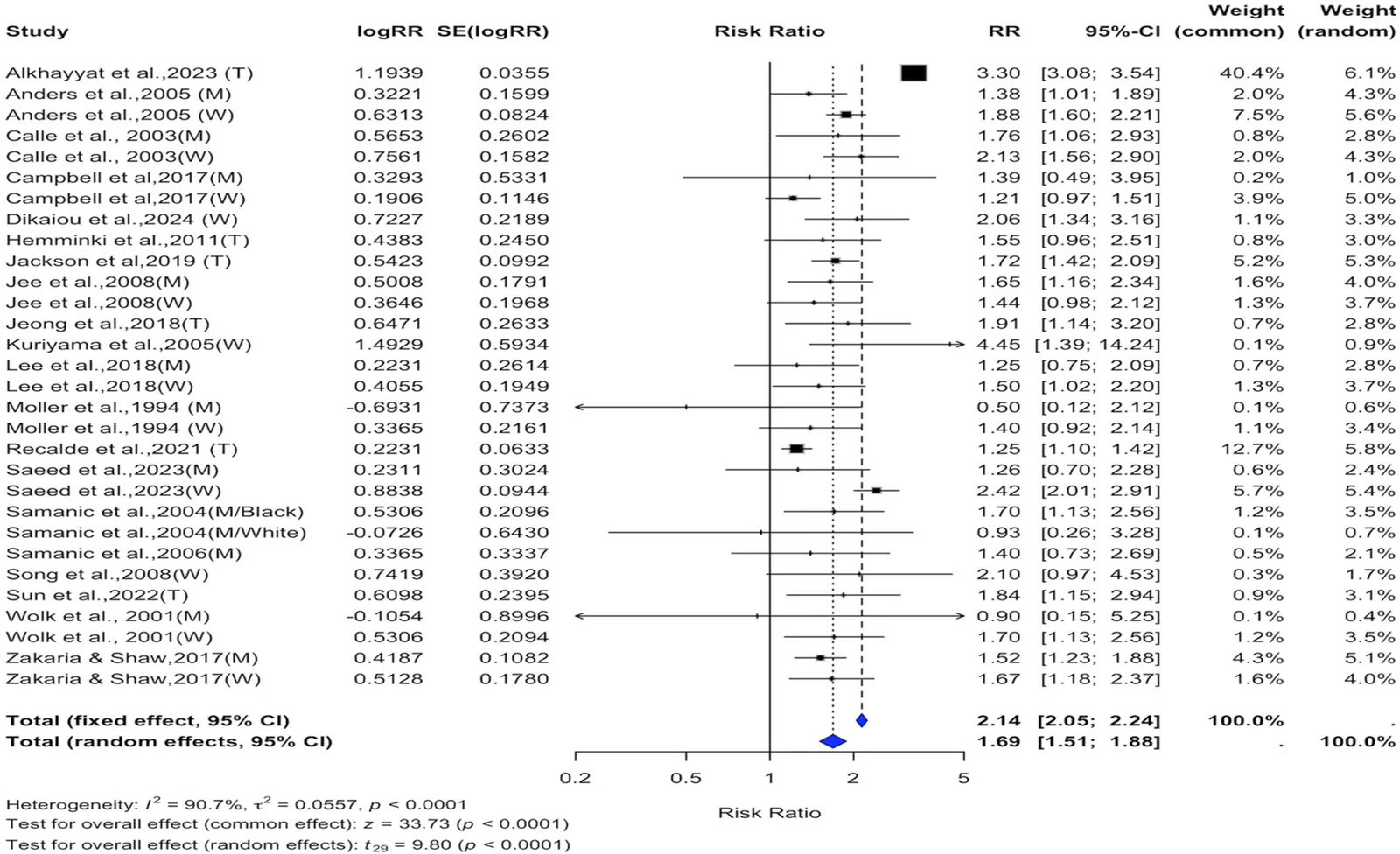
Relative risks of gallbladder cancer associated with BMI. Forest plot of the risk of GBC associated with obesity (≥ 30 kg/m2) in the general population. M: Men, W: Women, T: Total

30 reports from 20 studies indicated an association between GBC risk and obesity. As shown in Fig. 8, the forest plot presents a meta-analysis synthesizing data on this association. The pooled RR under the fixed-effects model was markedly elevated at 2.14 (95% CI: 2.05–2.24), while the random-effects model yielded a slightly attenuated yet significant RR of 1.69 (95% CI: 1.51–1.88). These findings indicate that obese individuals have a 69–114% higher risk of developing GBC compared with those of normal BMI. Substantial heterogeneity was observed across studies (I^2^ = 90.7%, *p* < 0.0001), suggesting considerable inter-study variability. Therefore, a random-effects model was adopted for the primary analysis. The overall effect remained highly significant in both models (z = 33.73, *p* < 0.0001 for the fixed-effects model; t = 9.80, *p* < 0.0001 for the random-effects model).

### Subgroup analyses

**Fig. 9:**
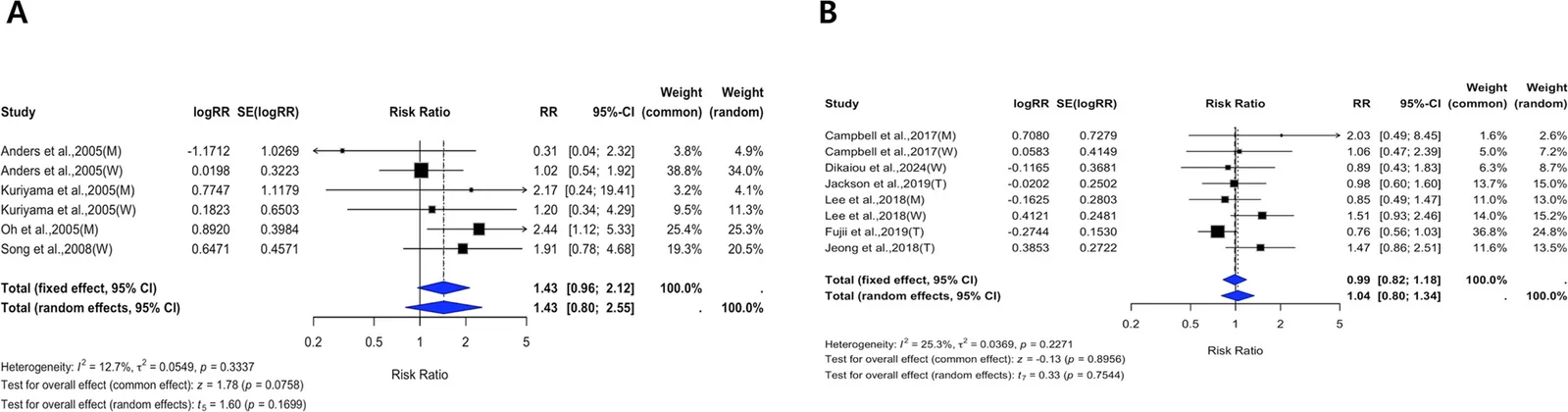
Comparison of underweight groups in previous studies and newly selected studies. **A** Previous studies, **B** Newly selected studies. M: Men, W: Women, T: Total

**Fig. 10:**
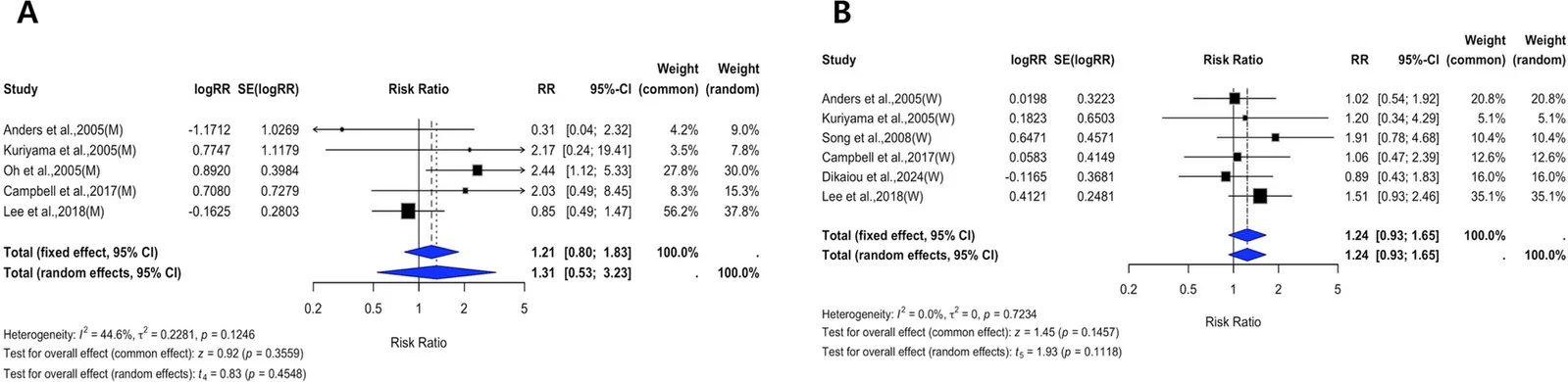
Comparison of underweight groups based on sex. **A** Men's group, **B** Women's group

In the underweight comparison, previous studies (Fig. 9A) showed a pooled RR of 1.43 (95% CI: 0.80–2.55) under the random-effects model and 1.43 (95% CI: 0.96–2.12) under the fixed-effects model. Heterogeneity was low (I^2^ = 12.7%), and the fixed-effects model was selected for the primary analysis. Newly included studies (Fig. 9B) reported a pooled RR of 1.04 (95% CI: 0.80–1.34) under the random-effects model and 0.99 (95% CI: 0.82–1.18) under the fixed-effects model. Heterogeneity was low (I^2^ = 13.8%). Therefore, it was decided that fixed-effects would be used in the results analysis. The direction of effect differed between earlier and more recent evidence—previous studies suggested a possible increased risk, while newer studies leaned toward a lowered risk. However, both comparisons lacked statistical significance, and CIs overlapped considerably, indicating no consistent evidence that underweight status influences GBC risk across study periods. Men group with underweight (Fig. 10A) showed a pooled RR of 1.31 (95% CI: 0.53–3.23) under the random-effects model and 1.21 (95% CI: 0.80–1.83) under the fixed-effects model. Heterogeneity was moderate (I^2^ = 44.6%), and the fixed-effects model was selected for the primary analysis. Women group with underweight (Fig. 10B) reported a pooled RR of 1.24 (95% CI: 0.93–1.65) under the random-effects model and 1.24 (95% CI: 0.93–1.65) under the fixed-effects model. There was no heterogeneity (I^2^ = 0.0%). Therefore, it was decided that fixed-effects would be used in the results analysis. Both groups showed a potential, possibly increased risk, but none were statistically significant.

**Fig. 11:**
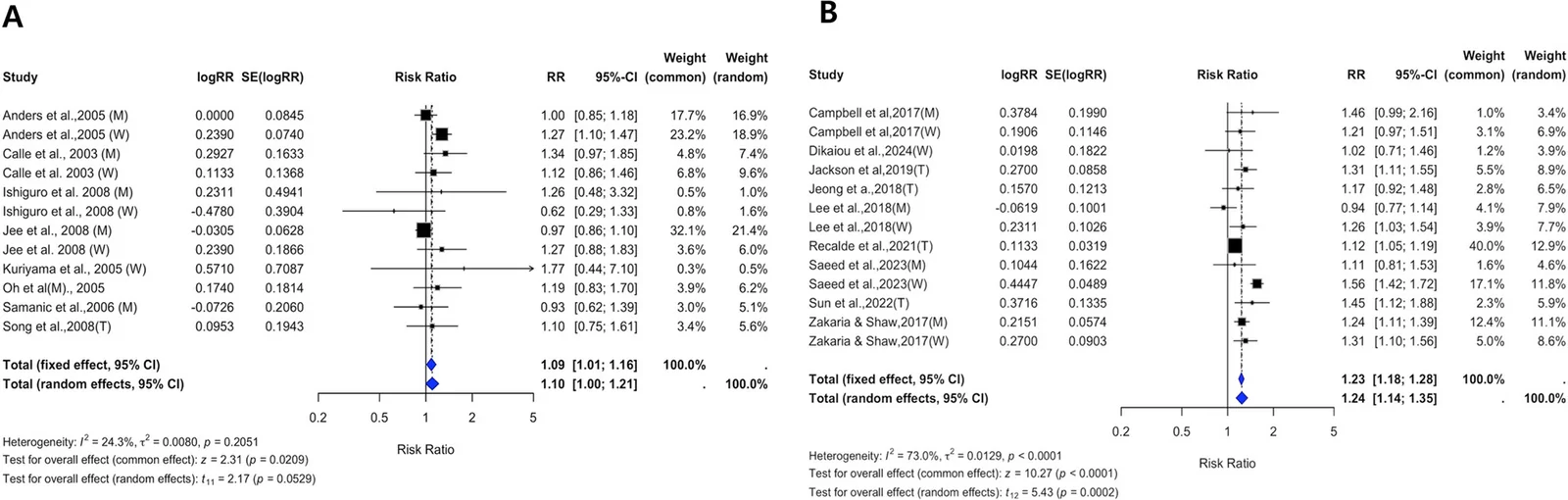
Comparison of overweight groups in previous studies and newly selected studies. **A** Previous studies, **B** Newly selected studies. M: Men, W: Women, T: Total

**Fig. 12:**
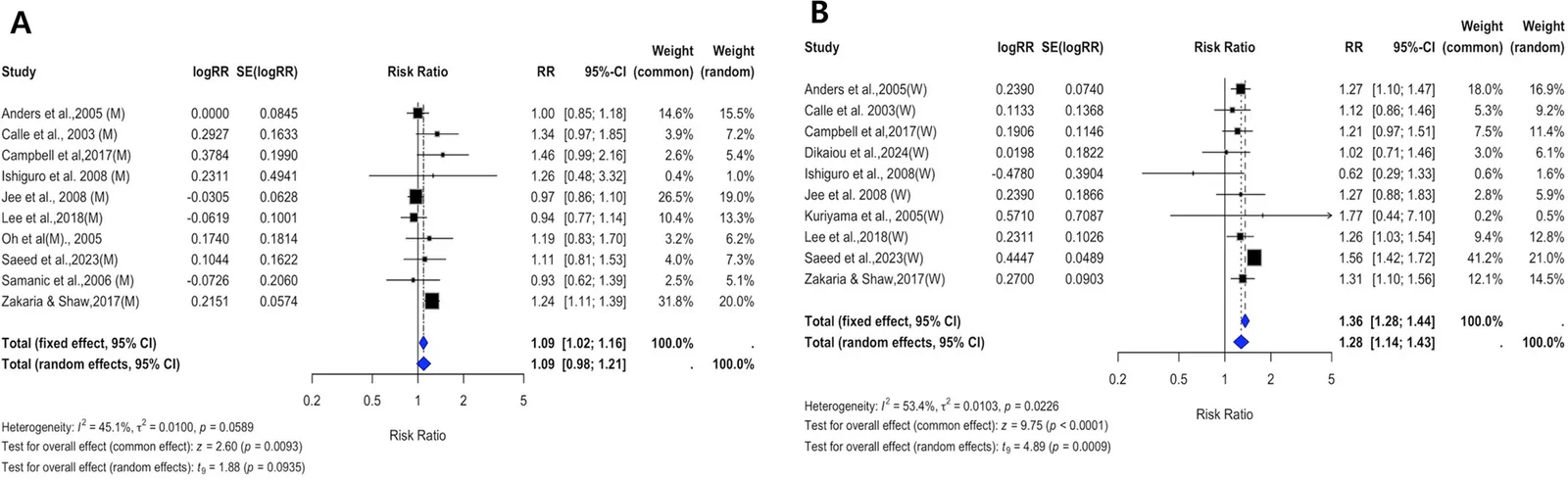
Comparison of overweight groups based on sex. **A** Men's group, **B** Women's group

In the overweight comparison, previous studies (Fig. 11A) showed a pooled RR of 1.10 (95% CI: 1.00–1.21) under the random-effects model and RR of 1.09 (95% CI: 1.01–1.16) under the fixed-effects model, suggesting a borderline significant association with increased GBC risk. Heterogeneity was low (I^2^ = 24.3%), and the fixed-effects model was selected for the primary analysis. In contrast, newly included studies (Fig. 11B) demonstrated a stronger and statistically significant association, with a pooled RR of 1.24 (95% CI: 1.14–1.35) accompanied by high heterogeneity (I^2^ = 73.0%) in the random-effects model, and RR of 1.23 (95% CI: 1.18–1.28) in the fixed-effect model. Accordingly, the random-effects model was adopted. The direction of effect was consistent across both groups, but the association was larger and more robust in the newly included studies, suggesting stronger evidence linking overweight status to elevated GBC risk in recent research. Men group with overweight (Fig. 12A) showed a pooled RR of 1.09 (95% CI: 0.98–1.21) under the random-effects model and 1.09 (95% CI: 1.02–1.16) under the fixed-effects model. Heterogeneity was moderate (I^2^ = 45.1%), and the fixed-effects model was selected for the primary analysis. Women group with overweight (Fig. 12B) reported a pooled RR of 1.28 (95% CI: 1.14–1.43) under the random-effects model and 1.36 (95% CI: 1.28–1.44) under the fixed-effects model. Heterogeneity was moderate to high (I^2^ = 53.4%). Therefore, it was decided that random-effects would be used in the results analysis. Even though both groups exhibited a clear association with GBC risk, the association was larger in the women’s group. It proved stronger evidence linking women to elevated GBC risk in overweight status than men.

**Fig. 13:**
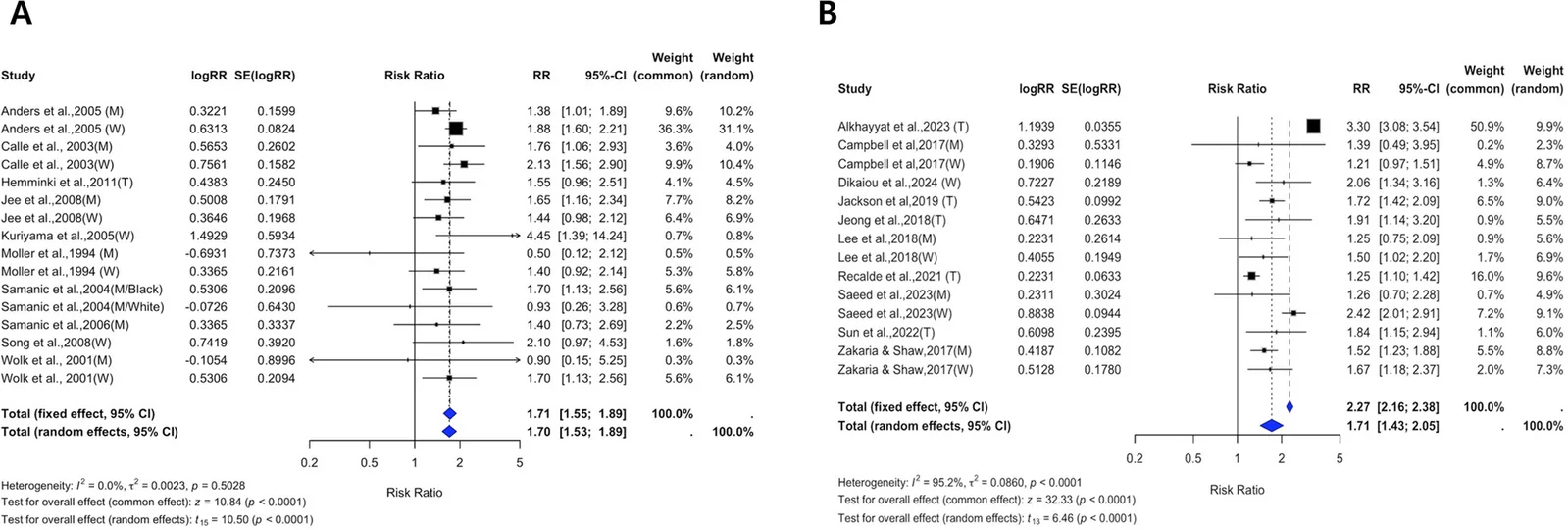
Comparison of obese groups in previous studies and newly selected studies. **A** Previous studies, **B** Newly selected studies. M: Men, W: Women, T: Total

**Fig. 14:**
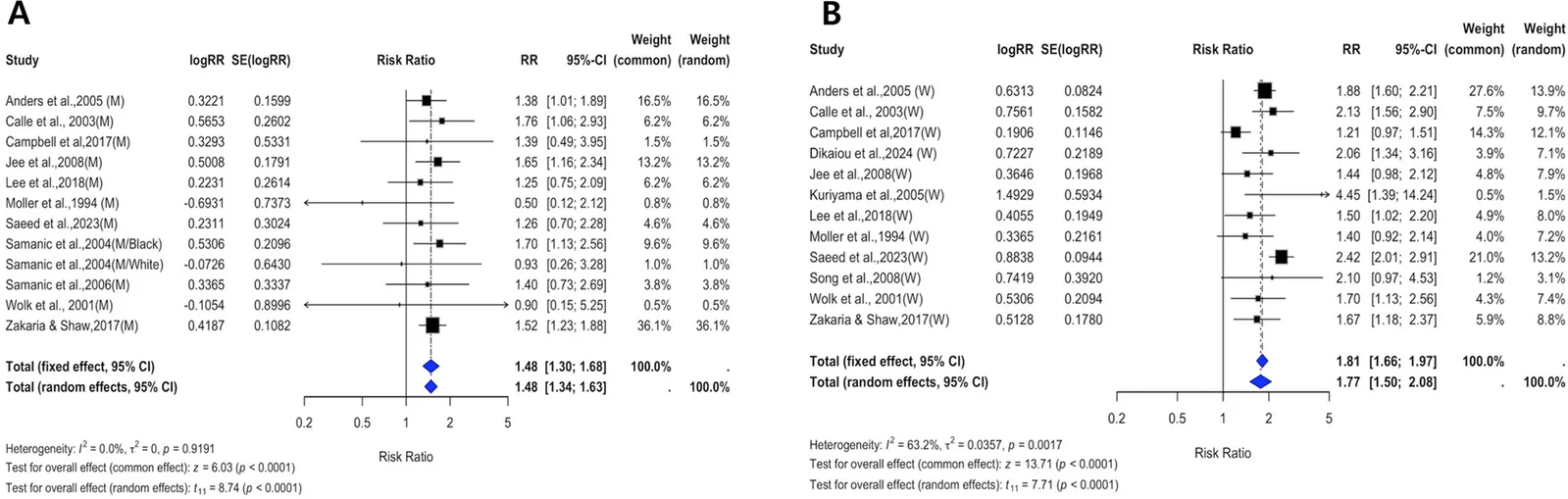
Comparison of obese groups based on sex. **A** Men's group, **B** Women's group

In the obesity comparison, previous studies (Fig. 13A) reported a pooled RR of 1.70 (95% CI: 1.53–1.89) under the random-effects model and 1.71 (95% CI: 1.55–1.89) under the fixed-effects model, showing a significant positive association with no observed heterogeneity (I^2^ = 0.0%). The fixed-effects model was therefore applied. Newly included studies (Fig. 13B) also demonstrated a significant association, with a pooled RR of 1.71 (95% CI: 1.43–2.05) under the random-effects model and 2.27 (95% CI: 2.16–2.38) under the fixed-effects model, accompanied by very high heterogeneity (I^2^ = 95.6%). The random-effects model was therefore selected for the primary analysis. Although the pooled effect size was similar across older and newer studies, the newer studies exhibited greater variability in individual estimates. Overall, both sets of studies consistently indicated that obesity is associated with a markedly increased risk of GBC. Men group with obese (Fig. 14A) showed a pooled RR of 1.48 (95% CI: 1.34–1.63) under the random-effects model and 1.48 (95% CI: 1.30–1.68) under the fixed-effects model. Heterogeneity didn’t exist (I^2^ = 0.0%), and the fixed-effects model was selected for the primary analysis. Women group with overweight (Fig. 14B) reported a pooled RR of 1.77 (95% CI: 1.50–2.08) under the random-effects model and 1.81 (95% CI: 1.66–1.97) under the fixed-effects model. Heterogeneity was high (I^2^ = 63.2%). Therefore, it was decided that random-effects would be used in the results analysis. Even though both groups exhibited a clear association with GBC risk, the association was larger in the women’s group. It proved stronger evidence linking women to elevated GBC risk in obese status than men.

## Discussion

The purpose of this meta-analysis was to update prior evidence and assess whether the association between excess body weight and GBC risk has changed over time. In addition, it aimed to evaluate the association between underweight and GBC risk, which has not been adequately addressed in previous meta-analyses. The study results showed that underweight individuals had a 5% higher RR compared with individuals of normal weight, despite no statistical significance. Even the leave-one-out analysis identified Fujii et al. [44] as a highly influential study in the underweight subgroup. This study contributed the largest statistical weight and reported an effect estimate below unity, thereby attenuating the overall pooled association. When this study was excluded, the pooled estimate increased and heterogeneity was eliminated (RR = 1.22, 95% CI: 1.00–1.48, I^2^ = 0%), suggesting a more consistent positive association across the remaining studies. This pattern indicates that the inclusion of this single influential study may obscure an underlying relationship between underweight and gallbladder cancer risk. Similar findings have been reported in other malignancies. For example, one study found that the risk of gastric cancer was 12.4% higher in underweight individuals compared with those of normal weight [51], a result comparable to the 15.5% higher risk of gastric cancer observed in overweight individuals [52]. Other studies have reported that in childhood, underweight increases cancer mortality by 285–315% [53]. In the present study, the underweight patients also presented with more aggressive tumors, as evidenced by higher tumor marker levels compared with patients having normal weight or overweight [53]. This finding suggests that underweight status may reflect frailty and low lean mass, both of which contribute to vulnerability to life-threatening illnesses. Low lean mass implies limited nutrient reserves, which compromises the ability of the body to tolerate illness and reduces the available treatment options. Furthermore, previous studies have reported that poor nutritional status is associated with increased treatment toxicity and reduced response to anti-cancer therapy [54–56]. In contrast, overweight and obese patients may benefit from greater nutritional reserves and have been shown to experience lower rates of treatment-related toxicity, whereas underweight patients have higher toxicity rates [53]. Collectively, these findings suggest that the adverse impact of underweight observed in other cancers may also apply to patients with GBC, contributing to poor prognosis and higher RR compared with individuals of normal weight.

However, studies directly examining the correlation between underweight and GBC risk remain rare, making it difficult to establish a definitive relationship. Furthermore, in the subgroup analysis, the previous studies subgroup showed an RR of 1.43 (95% CI: 0.80–2.55), whereas the newly selected studies subgroup showed an RR of 0.97 (95% CI: 0.75–1.27). Despite the possible potential association between being underweight and the risk of GBC, the inconsistency in the association revealed by subgroup analyses has the possibility of negating the association. The inconsistent associations observed in the subgroup analyses may be explained by limited statistical power and the presence of wider confidence intervals within each subgroup. Because each subgroup contains a relatively small number of studies, the estimates are less precise and more susceptible to random variation, making it difficult to detect a statistically significant effect. When the subgroups are combined, the overall sample size increases substantially, which improves statistical power, narrows confidence intervals, and reduces the influence of random error. This allows a modest positive association between underweight status and gallbladder cancer risk, despite still not being statistically significant. Separately, further research will be required to determine whether the observed differences in the association between underweight and GBC risk, specifically between previous studies and the newly selected studies, stem from methodological differences or from the presence of clinical factors that may have mitigated the risk for GBC patients with underweight in the previous versus the newly selected studies.

Overweight individuals demonstrated a 19% higher RR for GBC compared with those of normal weight. In obesity, individuals showed a 69% higher RR compared with normal-weight individuals. Notably, statistical analyses for overweight and obesity demonstrated high heterogeneity, at 66.3% and 91.0%, respectively. The high heterogeneity for the obese group appears to be driven by several studies [29, 45, 47–50] that reported statistically significant increased risk. The largest effect size was observed in Saeed et al. [48], with an RR of 1.56 (95% CI: 1.42–1.72). In contrast, studies such as those by Ishiguro et al. [34] and Samanic et al. [30] reported reduced or non-significant risks, contributing to the observed variability. A similar pattern was noted for obesity. For example, Alkhayyat et al. [41] reported the largest effect size (RR = 3.30, 95% CI: 3.08–3.54). Other studies with strong associations included Kuriyama et al. [32](RR = 4.45), Saeed et al. [48] (RR = 2.42), and Zakaria & Shaw [50] (RR = 1.67). Conversely, studies such as Moller et al. [27] and Wolk et al. [28] reported non-significant or imprecise estimates with wide CIs, contributing minimally to the pooled effect. Furthermore, in the subgroup analysis, considering that the heterogeneity was 0.0% in the obese group among previous studies, compared to 95.2% in the newly selected studies, and that the heterogeneity was 0.0% in the men group versus 63.2% in the female group, it appears that methodological differences between the previous and newly selected studies, as well as sex, served as driver of this substantial heterogeneity. Similarly, in the subgroup analysis of the overweight group, the newly selected studies exhibited markedly higher heterogeneity (73.0%) than the previous studies (24.3%); likewise, regarding gender, women displayed high heterogeneity (53.4%) while men displayed moderate heterogeneity (45.1%). Consequently, the overweight group, despite exhibiting lower heterogeneity than the obese group, still displayed a high heterogeneity due to factors such as sex and methodology differences. In contrast, the heterogeneity of the underweight group is 27.3%, which is markedly lower than that of the overweight or obese groups. This relative homogeneity likely reflects the generally consistent direction and magnitude of effect estimates across the included studies. Furthermore, subgroup-specific forest plot analyses indicated that heterogeneity remained low across most strata, with the exception of the subgroup comprising underweight men, where variability was more pronounced. The overall consistency in findings may suggest a more uniform underlying biological response or reduced variability in exposure classification within this group.

A subgroup analysis comparing studies from previous meta-analyses with the newly included studies in this review yielded notable findings. For underweight individuals, earlier studies reported an RR of 1.43, whereas recent studies reported a lower RR of 0.99. For overweight individuals, previous studies reported RRs of 1.09, while the newer studies showed higher RRs of 1.24. A similar pattern was observed for obesity: the pooled RR of earlier studies was 1.71, while that of the newer studies was 1.71. Although the point estimates were comparable, the upper bound of the CI was higher in newer studies (2.05 vs. 1.89), suggesting greater variability in more recent evidence. A subgroup analysis based on sex, the men’s and women’s groups within the underweight range reported RRs of 1.21 and 1.24, respectively. For overweight individuals, the men’s group reported RRs of 1.09, while the women’s group reported RRs of 1.28. For obese individuals, the men’s group reported RRs of 1.48, compared to the women’s group reporting RRs of 1.77.

The comparatively elevated pooled risk estimates observed in newer studies may reflect underlying differences in the anthropometric and metabolic characteristics of the overweight strata. Specifically, more recent datasets may include populations with a higher prevalence and severity of overweight and obesity. The total number of cases in the earlier studies was 5,260, averaging 375.71 per study, whereas the newly included studies involved 10,330 cases, averaging 1,033 per study, demonstrating more than twofold increase in sample size. From an evolutionary perspective, obesity has been linked to adaptive mechanisms such as the thrifty gene and fructose survival hypotheses [57]. These traits were advantageous in environments characterized by intermittent food availability but have become maladaptive in modern societies. The rapid shift to “obesogenic” environments has outpaced genetic evolution, creating a mismatch between conserved human physiology and current conditions [58]. In industrialized settings, the widespread availability of calorie-dense, hyperpalatable foods, combined with reduced physical activity, promotes chronic positive energy balance. Additional factors, including circadian disruption, environmental stressors, and socioeconomic constraints, further impair metabolic regulation. These influences disrupt hormonal pathways such as leptin and insulin signaling and alter gut–brain communication, leading to impaired satiety and increased energy intake [59, 60]. Over time, these processes contribute to visceral adiposity, chronic inflammation, and metabolic dysfunction. Thus, the interaction between evolutionary adaptations and modern environments plays a central role in the rising global prevalence of overweight and obesity [60]. Consequently, the interplay of lifestyle, dietary, genetic, and evolutionary factors in modern society has contributed to the rapid rise in overweight and obesity prevalence, as reflected in recent studies reporting a higher number of individuals with abnormal body weight.

The finding that women exhibit a higher risk of gallbladder cancer (GBC) than men across all abnormal weight categories—underweight, overweight, and obesity—provides important clinical insight. A previous meta-analysis reported that women with overweight or obesity had a higher risk of GBC compared to men, while the association in men remained inconclusive [18]. The present analysis not only confirms the elevated risk among overweight and obese women but also suggests that this increased susceptibility may extend to underweight individuals. Moreover, unlike prior findings, a positive association between overweight status and GBC risk was also observed in men, indicating a broader relationship between abnormal body weight and GBC risk across sexes.

A biologically plausible explanation for the higher risk observed in women involves the role of estrogen signaling. Estrogen receptor beta (ERβ) has been reported to be highly expressed in GBC, with one study identifying its presence in approximately 89% of cases and associating it with poorer survival outcomes. This suggests a potential role for estrogen-mediated pathways in GBC pathogenesis and progression. In the context of obesity, adipose tissue functions as an active endocrine organ and a major site of peripheral estrogen production. Elevated estrogen levels may increase cholesterol saturation in bile and impair gallbladder motility, thereby promoting gallstone formation and chronic inflammation—well-established precursors to GBC [61]. In addition, estrogens play a critical role in regulating energy balance and metabolic homeostasis. Reduced estrogen signaling has been associated with decreased energy expenditure and increased susceptibility to weight gain [62]. These observations highlight a complex and potentially bidirectional relationship between estrogen, adiposity, and carcinogenesis. On one hand, increased estrogen exposure in obesity may enhance tumor-promoting pathways; on the other, reduced estrogen activity may contribute to metabolic dysfunction and obesity, indirectly elevating cancer risk. This interplay underscores a biologically intricate framework in which hormonal regulation, metabolic state, and oncogenic processes are closely interconnected. Collectively, these findings suggest that sex-specific hormonal mechanisms, particularly those involving estrogen signaling, may partially explain the higher susceptibility to GBC observed in women. The convergence of endocrine, metabolic, and inflammatory pathways may amplify risk in female populations, contributing to the observed sex disparity across BMI categories.

As a result, this meta-analysis provides several valuable insights. First, it is the first to suggest a possible association between GBC and underweight. Second, by incorporating recently published studies, it reflects updated trends in GBC risk among obese individuals. Third, through subgroup analysis comparing older and more recent studies, it shows that the impact of excess body weight on GBC risk appears more pronounced in recent years. Fourth, subgroup analysis based on sex indicates that women exhibit a higher risk of GBC than men. Above all, the fact that no significant bias was detected in the publication bias analysis, and that the results were not unduly influenced by any specific study, as demonstrated by the sensitivity and leave-one-out analyses, attests to the robustness of this meta-analysis.

Nonetheless, some limitations should be acknowledged. First, statistical analysis showed no significant association between underweight and GBC risk. Second, reliance on BMI as the exposure measure has inherent shortcomings. While BMI is a convenient screening tool, it is not a direct measure of adiposity and lacks accuracy in reflecting true fat mass at the individual level. BMI may overestimate adiposity in athletes with high muscle mass or in patients with edema, and underestimate adiposity in sarcopenic individuals with low lean mass [63–66]. This potential misclassification could distort case categorization and, consequently, bias the pooled effect estimates. Another important limitation relates to the variability in follow-up duration across the included studies, which ranged from 5 to 28 years. Studies with shorter follow-up periods may be more susceptible to reverse causation, whereby preclinical or undiagnosed gallbladder cancer leads to unintentional weight loss prior to diagnosis, resulting in individuals being classified as underweight at baseline. This phenomenon could artificially inflate the observed association between underweight status and gallbladder cancer risk. In contrast, studies with longer follow-up durations are less likely to be affected by this bias, as baseline BMI is more likely to reflect habitual body weight rather than disease-related changes. Finally, the quality of included studies warrants caution. Among the newly included studies, ROBINS-I v2 quality assessment indicated that only eight of 25 were rated as “low” risk of bias. Although none were rated as “critical,” 15 were assessed as “moderate” and one as “serious,” suggesting possible residual bias or inadequate confounder adjustment. However, the first limitation holds potential for resolution, as a leave-one-out analysis revealed that the study by Fujii et al. [43] disrupted the association between underweight status and GBC risk.

## Conclusion

In conclusion, consistent with previous studies, this meta-analysis confirmed that the risk of GBC increases with higher body weight. When comparing more recent studies with earlier ones, the risk of GBC associated with excess body weight was higher in recent years, suggesting that obesity- and overweight-related cancer risk may continue to rise in the future. It was also observed that the tendency for women to have a relatively higher risk of GBC than men persisted. Additionally, this meta-analysis identified a potential, possibly increased GBC risk in the underweight group. Although the underlying mechanisms could not be determined, this finding is supported by indirect evidence from studies on other cancers. However, there was no statistical significance that the revealed higher risk may have emerged merely by statistical chance. Future research should therefore not only monitor the increasing burden of GBC linked to excess body weight but also investigate the biological mechanisms underlying the association between underweight and GBC risk.

## Supplementary Information

Supplementary Material 1.

## Data Availability

All data generated or analyzed during this study are included in this published article.

## Abbreviations

GBC: Gallbladder Cancer
WHO: World Health Organization
BMI: Body Mass Index
MOOSE: Meta-analysis Of Observational Studies in Epidemiology
RR: Risk Ratio
OR: Odds Ratio
HR: Hazard Ratio
CI: Confidence Interval
Robins-I: Risk of bias in non-randomized studies of interventions
